# Commentary: The desire of medical students to integrate artificial intelligence into medical education: An opinion article

**DOI:** 10.3389/fdgth.2023.1151390

**Published:** 2023-04-06

**Authors:** Jimmy Y. Zhong, Nastassja L. Fischer

**Affiliations:** ^1^ Science of Learning in Education Centre (SoLEC), Office of Education Research, National Institute of Education, Nanyang Technological University, Singapore, Singapore; ^2^ Center for Advanced Brain Imaging (CABI), Georgia Institute of Technology, Atlanta, GA, United States; ^3^ Centre for Research and Development in Learning (CRADLE), Nanyang Technological University, Singapore, Singapore

**Keywords:** artificial intelligence, machine learning - ML, technical skill building, medical education - clinical skills training, medical ethics

A Commentary on The desire of medical students to integrate artificial intelligence into medical education: An opinion article By Frommeyer TC, Fursmidt RM, Gilbert MM and Bett ES. (2022) Front. Digit. Health 4:831123. doi: 10.3389/fdgth.2022.831123

## Introduction

Artificial intelligence (AI) is widely regarded as the suite of technologies that allow computers to perform tasks that resemble human abilities (e.g., speech and pattern recognition, rendering advice, generating new contents, etc.) (1, 2). It has proven its merits in the healthcare industry over the past decades and there are promising prospects that it will continue to do so in the years to come (3, 4). The recent opinion article by Frommeyer et al. (5) highlighted the value of AI for the medical profession with respect to precision medicine, drug discovery, disease diagnosis, and healthcare management, before making a “call to action” for the introduction of “guided seminars and courses on biostatistics, digital health literacy, and engineering technologies (5, p. 3).” In this commentary, we respond to this call for AI-oriented medical education by providing some internationally applicable ideas that would turn this proposal of Frommeyer's et al. (5) into reality.

## AI knowledge that matters

### Computational and programming procedures

Frommeyer et al. (5), and more recently Ravindran (4), gave some convincing arguments for the practical benefits of using AI tools, which operate on the basis of machine learning (ML), in the provision of certain healthcare services. These advantages relate to the enormous savings in time and human resources when conducting (i) radiological imaging (i.e., optimizing screening of potentially cancerous tissues during oncological examination), (ii) discovery of new drugs by using large datasets containing information about the chemical and molecular constituents of pre-existing drugs, and (iii) creating therapeutic and treatment regiments that are personalised according to the patient's needs [for details, see (4–6)]. Even though these AI tools generated lots of attention from healthcare professionals and researchers due to their high efficiency in delivering tangible outcomes; we believe that it would be more valuable if the focus turns to giving future medical and healthcare professionals a technical and holistic understanding of AI-based computational and programming processes directed to the provision of specific healthcare services. Accordingly, we do not see it as enough for medical schools to instruct their students on the basic and non-hands-on facts of AI methods only (i.e., telling medical students what AI is and what it can do without informing them about some details of the computational processes involved). In this sense, we do not want future medical practitioners and researchers to become mere consumers or “regurgitators” of AI-related content knowledge. Instead, we want such professionals to learn and think critically about how AI works, so they can use it properly in their careers with an informed mindset. For instance, if students were to be taught how AI facilitates the discovery of new drugs for treating cancer, they should understand where the data comes from, its qualitative and quantitative features, the general category of the AI algorithm (e.g., supervised, unsupervised, or reinforced learning) and the specific type of AI method to be used [e.g., decision trees, k-nearest neighbor (KNN), convolutional neural network (CNN)], how to partition the data and cross-validate the results by using training and testing datasets, and perhaps most importantly, how to analyze and interpret the results with respect to the research questions at hand. The successful final interpretation of computational findings would require the domain-specific knowledge of a medical/healthcare professional, as well as the technical expertise of a computer scientist or engineer who is skilled in AI/ML matters. As such, an interdisciplinary and collaborative effort is crucial – with the medical expert knowing the general pattern of findings that needs to be attained in order to answer the research questions on one hand – and the computing/engineering expert knowing the functions of the algorithms deployed, how the statistical outputs were generated, and what they mean on the other. With this in mind, we recommend an educational partnership between medical doctors and computer scientists/engineers in the form of serving as instructors in the medical-themed AI/ML program proposed herein. Ideally, the two parties should also communicate openly and pool their expertise together when developing new forms of AI/ML algorithms or tools for tackling specific medical research problems.

Taking a broader perspective, the acquisition of all these technical know-how mentioned above by medical students would be best served by them having some background programming knowledge and experience with coding AI/ML functions. However, owing to the fact that programming/coding skills are the mainstay of computer scientists and engineers, and not characteristic of the average medical student or healthcare professional, we advocate the use of ML software packages with intuitive graphical user interfaces that operate on the basis of drop-down menus and point-and-click functions. Currently, KNIME is prime example.^1^ It is an open-source software with a studio-like user interface that allows users to build, visualize, and deploy ML models through the construction of nodes-and-edges flowcharts. KNIME can be learned easily through online video courses (for instance, many video tutorials are freely available on YouTube), and we recommend medical instructors to consider KNIME as a serious candidate for their hands-on ML tutorials and workshops.

### Caveats against the “Omnipotence” of AI

In addition to learning about the nuances of AI/ML operations, we would like to stress that these technical courses should be complemented by a firm conceptual understanding of what AI can and cannot do in providing healthcare services. For example, Kompa et al. (7) highlighted the importance of quantifying and communicating the probabilistic concept of uncertainty or stochasticity when using ML techniques for medical diagnostics and predictions. We see this approach as important because it enables healthcare professionals to become more perceptive when using AI and minimizes the risks of them making overestimated or unrealistic interpretations of findings or outputs derived from the use of AI/ML tools. We believe that the delivery of such knowledge to the medical/healthcare community is crucial because we do not want our proposed AI medical curriculum to be a mere copy of courses offered by a typical computer science or engineering department – that is, one that focuses primarily on programming and statistics without due considerations of the limitations of AI technology in healthcare and the human factors involved. Specifically, we do not want attendees of such an AI medical program to enter and leave with a “one-track mind,” thinking that AI is the “be-all” and “end-all” for human technological progress. This statement is of utmost importance given the presence of some overly optimistic present-day opinions favoring the use of AI tools in medical practice (4), biases that can potentially lead to flawed conclusions by the general public – for instance, that AI will eventually replace human radiologists and pathologists simply due to its higher accuracy in medical image analysis (4). We want to caution against the espousal of such views because other AI experts have opined that AI algorithms work best for medical imaging tasks that are specific and narrowly defined [e.g., identifying chest nodules and brain hemorrhages (3),]. This relates to the fact that many present-day AI/ML models are limited by an inability to extrapolate beyond the types of data used to validate them in the first place (8), meaning that a trained AI model works best with respect to dataset features that are congruent with or similar to those in the original training dataset, and that it cannot be applied flawlessly to perform predictions or inferences using datasets with features that are largely discrepant from those present in the training dataset. For instance, while AI can outperform human pathologists in the accurate detection of bone fractures and internal bleeding, it performs much poorer than humans in interpreting brain images from patients with acute neurological disorders (9). Evidence of the latter sort shows that although AI methods can serve as helpful tools, they are still far from replacing human critical thinking and inferential skills anytime soon. We will still need medical doctors around to interact with patients, to inform them about the nature of their illnesses in a congenial fashion, and to perform medical image-guided interventions (3). These are the “ingredients” of “human touch” that AI cannot replace.

### “Black box” nature of conventional AI/ML models

Following this trend of thought, we also deem it important to inform medical students about the so-called “black box” nature of most AI/ML algorithms/models today, which relates to their inabilities to show human users how they computed their decisions and generated their outputs (10). Currently, there is a movement to overcome the impediment posed by non-explainable, black box-like AI/ML models through the development of advanced explainable AI (XAI) techniques [e.g., LIME and SHAP, see (10), for descriptions and formulas], which are set to offer enormous benefits to industries such as aviation and medical healthcare by making the decision-making process of the AI/ML algorithm more transparent to the user. This development, however, does not negate the fact that many of the most popular AI/ML models are “black boxes” by initial design (e.g., KNN, random forests, neural networks, support vector machines), and that the validity of their outputs are highly contingent on the quality of the input data (10). Notably, the input data can be affected by sampling or selection biases, which can create samples that are unrepresentative of the general population, culminating in findings that are not entirely dependable and ecologically valid. In other words, “black box” models, by not making the links between input and output data explicit to the user, requires the user to exert extra care in ensuring the quality and representativeness of the input data so as to ensure that the output findings carry merits for generalization and real-world application. Henceforth, we hope to see the inclusion of these vital facts concerning data selection/sampling and quality assurance in the medical curriculum proposed herein.

## AI-related Ethics

Moreover, owing to the fact that AI applications for medical and healthcare use is an emerging rather than an established technology, the laws, regulations, and policies governing its creation and implementation are still in a state of ongoing maturation (1, 11). Nevertheless, medical students, as well as current healthcare professionals, should not stay ignorant about the ethical and legal concerns surrounding AI use and governance. As many AI algorithms or programs rely on a large pool of data for ML purposes, medical students ought to be taught about the ethical principles involved in AI-related data collection, storage, and analysis, so as to insure the protection of the basic rights of human patients or subjects. In this respect, we deem it as important for medical instructors to inform their students about the regional and international conventions and charters governing AI use (11), and to encourage them to apply such knowledge when embarking on their medical careers. Even though teaching ethics may seem “easy” when compared with mainstream medical topics, we still recommend some systematic teaching efforts to be in place in order to forestall the risks of AI misuse by future medical practitioners.

## Closing statement

In conclusion, this article is meant to be a short commentary on some important considerations for the inclusion of AI content in medical schools' curriculum (see Table 1, for a summary of the main topics discussed above). We kept our ideas generic in nature in order to ensure freedom of choice for different medical colleges to work on the specifics of their implementation based on local academic norms. It is with our best wishes that these ideas, once implemented, will bring about higher quality education for future generations of medical students and healthcare professionals.

**Table 1:** Key considerations for an AI-oriented medical curriculum.

Summary List of Proposed Ideas
Computational and programming procedures of how AI works in specific healthcare applications. • Learning and executing ML techniques through user-friendly software with intuitive graphical user interfaces (e.g., KNIME). • Educational partnership between medical doctors and computer scientists/engineers in teaching AI/ML technical components. Concepts and facts that caution against the potential harms of endorsing overly positive views of AI in healthcare. • Probabilistic concepts/principles underlying ML techniques. • Inability of many current AI/ML models to extrapolate beyond the constraints of a given dataset used for model creation. • Sampling/Selection biases that can “contaminate” outputs derived from “black box” AI/ML models. Ethical considerations of AI use in healthcare services and associated legalities. • Understanding AI-related laws, regulations, and policies. • Ethical principles underlying AI data collection, storage, analysis. • Regional and international AI-related conventions and charters.

## Footnotes

^1^KNIME Analytics Platform. https://www.knime.com/
